# Copy number alteration of neuropeptides and receptors in multiple cancers

**DOI:** 10.1038/s41598-017-04832-0

**Published:** 2017-07-04

**Authors:** Min Zhao, Tianfang Wang, Qi Liu, Scott Cummins

**Affiliations:** 1 School of Engineering, Faculty of Science, Health, Education and Engineering, University of the Sunshine Coast, Maroochydore DC, Queensland, 4558 Australia; 2 Department of Biomedical Informatics, Vanderbilt University School of Medicine, Nashville, Tennessee 37232 United States; 3 Center for Quantitative Sciences, Vanderbilt University School of Medicine, Nashville, Tennessee 37232 United States

## Abstract

Neuropeptides are peptide hormones used as chemical signals by the neuroendocrine system to communicate between cells. Recently, neuropeptides have been recognized for their ability to act as potent cellular growth factors on many cell types, including cancer cells. However, the molecular mechanism for how this occurs is unknown. To clarify the relationship between neuropeptides and cancer, we manually curated a total of 127 human neuropeptide genes by integrating information from the literature, homologous sequences, and database searches. Using human ligand-receptor interaction data, we first identified an interactome of 226 interaction pairs between 93 neuropeptides and 133 G-protein coupled receptors. We further identified four neuropeptide-receptor functional modules with ten or more genes, all of which were highly mutated in multiple cancers. We have identified a number of neuropeptide signaling systems with both oncogenic and tumour-suppressing roles for cancer progression, such as the insulin-like growth factors. By focusing on the neuroendocrine prostate cancer mutational data, we found prevalent amplification of neuropeptide and receptors in about 72% of samples. In summary, we report the first observation of abundant copy number variations on neuropeptides and receptors, which will be valuable for the design of peptide-based cancer prognosis, diagnosis and treatment.

## Introduction

The nervous system is the superordinate structure in the body, controlling the functions and activities of virtually all other tissues and organs, including cancer tissues^1^. Neuropeptides are a group of signaling messengers that function as neurotransmitters, paracrine regulators, and hormones to regulate exocrine and endocrine secretion, smooth muscle contraction, blood pressure, and inflammation. Previously, neuropeptides have also been recognized as potent cellular growth factors for many cell types, including cancer cells^2^, however, the molecular mechanism of this relationship is unknown.

In general, G-protein coupled receptors (GPCRs) are the principal neuropeptide binding targets through which intracellular signaling transduction pathways are triggered^3^. GPCRs are composed of seven transmembrane domains that transduce the signal intracellular through G proteins^4^. They are found in almost all eukaryotes and have a diverse array of ligands ranging from light, Ca2 + and odorants, to small molecules such as amino acid residues, nucleotides, peptides, and proteins^4^. Accumulated studies on the signaling pathways activated by neuropeptide-GPCRs have revealed unsuspected connections and complexities^4^. For example, neuropeptides may interact with several rather than a single GPCR. Moreover, GPCRs not only stimulate the synthesis of conventional second messengers but also induce multiple pathways leading to tyrosine phosphorylation events^4^. GPCR signaling via G-protein-independent pathways (such as direct binding of Src to the receptors) has also been suggested to regulate cancer cell growth and malignant transformation^5^.

Despite a number of studies of neuropeptides and their GPCR receptors in different species^6, 7^, the global interaction of neuropeptides and GPCR receptors are unknown. Although neuropeptides have been used to improve biotherapy against Human pancreatic cancer^8^, the role of neuropeptides and their interacting receptors in cancers is unexplored. In this study, we created a comprehensive inventory of neuropeptides by searching all the known and putative mammalian neuropeptides. Also, we constructed the first human neuropeptide-receptor network. By overlapping with large-scale pan-cancer genomics data from The Cancer Genome Atlas (TCGA), we also conducted mutational and expression analysis to elucidate the relationship between neuropeptide and cancer development.

## Results

### The pan-cancer prognostic features of curated neuropeptides

**Figure 1:**
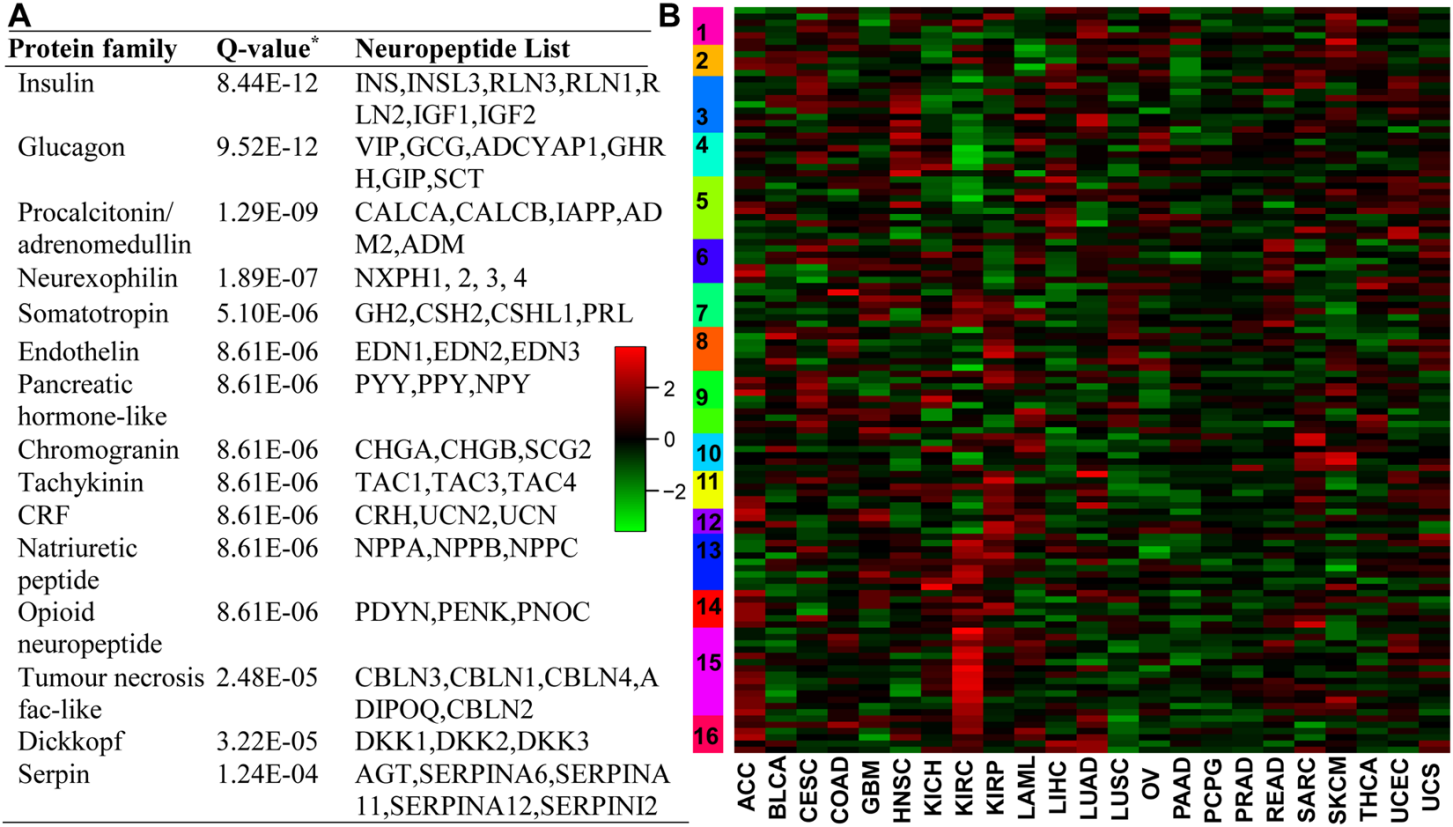
Overall features of neuropeptides. (**A**) The top 15 enriched protein families in 127 human neuropeptides. Note: *Q-values: the raw P-values of the hypergeometric test were corrected by Benjamini-Hochberg multiple testing correction. (**B**) Heatmap of prognostic Z-scores of 119 neuropeptides in the 23 TCGA cancer set. The prognostic Z-scores obtained from PRECOG database are represented by the scale bar. The polarity of the prognostic Z-score reflects the direction of association. The TCGA cancer types are abbreviated as ACC: Adrenocortical carcinoma; BLCA: Bladder Urothelial Carcinoma; CESC: Cervical squamous cell carcinoma and endocervical adenocarcinoma; COAD: Colon adenocarcinoma; GBM: Glioblastoma multiforme; HNSC: Head and Neck squamous cell carcinoma; KICH: Kidney Chromophobe; KIRC: Kidney renal clear cell carcinoma; KIRP: Kidney renal papillary cell carcinoma; LAML: Acute Myeloid Leukemia; LIHC: Liver hepatocellular carcinoma; LUAD: Lung adenocarcinoma; LUSC: Lung squamous cell carcinoma; OV: Ovarian serous cystadenocarcinoma; PAAD: Pancreatic adenocarcinoma; PCPG: Pheochromocytoma and Paraganglioma; PRAD: Prostate adenocarcinoma; READ: Rectum adenocarcinoma; SARC: Sarcoma; SKCM: Skin Cutaneous Melanoma; THCA: Thyroid carcinoma; UCEC: Uterine Corpus Endometrial Carcinoma; UCS: Uterine Carcinosarcoma.

To survey the potential role of neuropeptides in human cancers, we performed extensive data integration (see methods). We identified 127 human genes that encode neuropeptide precursors (Table S1). These precursor genes are usually processed to produce hundreds of neuropeptides by alternative splicing and post-translational modification. We classified these neuropeptides based on the protein family information (Fig. 1A) and found 15 families with three or more neuropeptides (P-values < 0.001). The largest family is insulin with seven genes: *INS*, *INSL3*, *RLN3*, *RLN1*, *RLN2*, *IGF1*, and *IGF2*.

To explore the potential prognostic application of these curated neuropeptides, we overlapped these genes with the human prognostic database PRECOG with survival outcomes^9^. We focused on the 23 TCGA datasets since these datasets were generated by the same data quality control and processing procedure. For each cancer set, PRECOG computed a Z-score to characterize the gene expression feature and clinical outcomes by minimizing the batch effects. In general, the polarity of the prognostic Z-score reflects the direction of survival association; a positive score reflects higher expression (adverse survival) and a negative score reflects lower expression (favorable survival). For our data there are 16 main clusters in the heatmap of PRECOG Z-score for the 23 TCGA cancer set (Fig. 1B). We assigned the cluster IDs in an order from top down. The majority of clusters are highly specific to one or two cancer types (Table S2). For example, the prognostic Z-scores of Cluster 15 are high in the Kidney Renal Clear Cell Carcinoma (KIRC). There are 13 genes out of 14 genes in the Cluster 15 with a prognostic Z-score greater than 1.96, which is equivalent to a two-tailed P < 0.05. In particular, there are five genes (*GNRH1*, *GNRH2*, *NPFF*, *NMU*, and *TAC3*) annotated with “Gastrin-CREB signalling pathway via PKC and MAPK” (Corrected P-value = 2.87E-05) and which could regulate glioblastoma (GBM) tumour cell proliferation by modulating the expression of three key cell cycle factors, cyclin B, D and proliferating cell nuclear antigen (*PCNA*). We also found that some neuropeptides from the same protein family cluster together in the prognostic heat map; these were *NXPH1*-*NXPH2*, *RLN1*-*RLN2*, and *EDN1*-*EDN2*. In summary, these distinct neuropeptide clusters that are specific to various cancer types provide the first insight into the potential prognostic significance of neuropeptide in specific cancers. Additionally, those neuropeptides from the same protein families may play a similar function in cancer progression and present similar prognostic features.

### The functional features of curated neuropeptides and receptors in human

To explore the literature-based neuropeptide receptor relationship, we have extracted a total of 260 neuropeptide-receptor pairs from a recently published human ligand-receptor network^10^. This resulted in a dataset containing 93 neuropeptides and 133 receptor genes in human (Table S3). We focused on those neuropeptides with known receptors for network reconstruction and the non-informatics neuropeptides were discarded because of a lack of receptor information.

**Figure 2:**
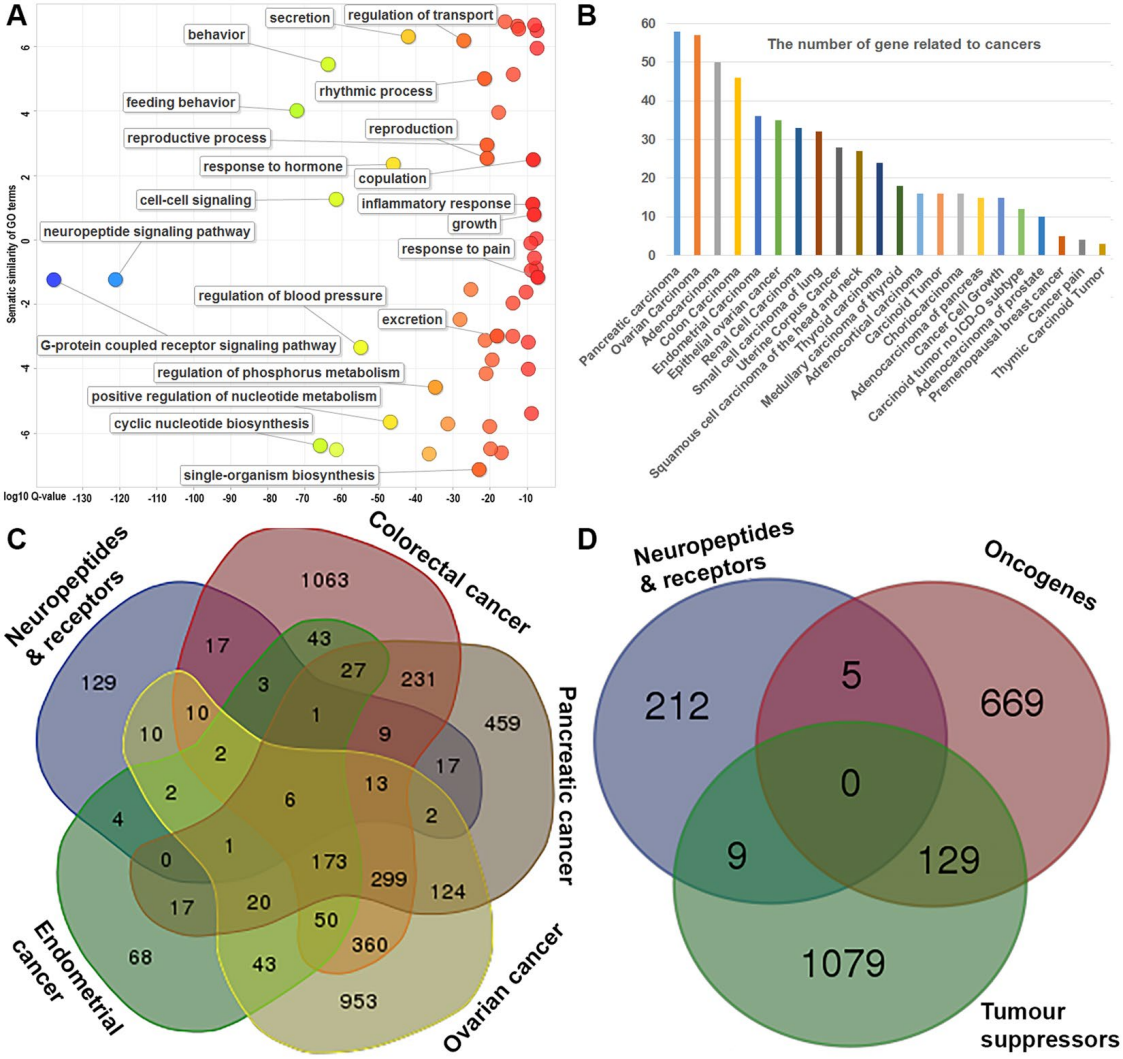
Functional analysis of the 93 neuropeptides and 133 receptors. (**A**) Scatterplot showing the biological process cluster representatives terms from gene ontology (GO) for the 127 neuropeptides in a two-dimensional space derived by applying multidimensional scaling to a matrix of the GO terms’ semantic similarities. Y-axis indicates the similarity of the GO terms; x-axis indicates the log of corrected P-value (bubbles of right corrected P-values are larger). (**B**) Graph showing significantly enriched cancer-related terms for 93 neuropeptides and 133 receptor genes in human. (**C**) Venn diagram showing comparative overlap based on four literature-based cancer databases. (**D**) Venn diagram showing overlap of tumour suppressors and oncogenes.

Further enrichment analyses were performed to obtain a functional overview for those 226 neuropeptide and receptor genes. As shown in Fig. 2, most of the gene ontology is related to neuropeptide and GPCR signalling pathways, as expected. Also, these genes are involved in many basic biological processes including regulation of blood pressure, inflammatory response, feeding behaviour, response to pain, copulation, reproduction, and rhythmic process (Fig. 2A). Interestingly, these neuropeptides and receptors are also the positive regulators of nucleotide metabolism, which itself may be related to DNA synthesis. The cellular component analysis revealed those neuropeptides and receptors that are mainly in the plasma membrane and extracellular space (Figure S1A). The significantly enriched molecular functions (Figure S1B) also confirmed their functions in peptide and receptor binding.

However, we also found that the 226 genes were associated with 22 different types of cancers based on the results of mining text within the DisGeNET database (Fig. 2B). The results also include some general terms related to cancer, such as adenocarcinoma and caner pain. The adenocarcinoma refers to those malignant tumors originating from glandular structures in epithelial tissue. Cancer pain is broadly caused by the tumor pressing on the bone, nerves and other organs in the body. Since various neuropeptides have roles in many human glands such as adrenal, saliva, and pituitary^11^, it was not surprising that neuropeptides have effects on adenocarcinoma growth. Similarly, cancer pain associated with neuropeptides has been reported previously^12^.

Interestingly, the cancers are closely related in terms of their anatomical location, including colon, ovary, pancreas, rectum, uterus, and endometrium. To confirm the oncogenic role of these neuropeptides and receptors, we intersected our 226 genes with four manually-curated cancer type specific databases^13–16^. As shown in Fig. 2C, we found six genes, *ADIPOQ*, *DKK1*, *IGF1*, *IGF1R*, *IGF2*, and *PLAUR* were shared by all the four cancers. By overlapping with known oncogenes and tumour suppressors^17^ (Fig. 2D), we found five oncogenes (*GALR2*, *IGF2*, *IGF1R*, *MAS1*, and *TAC1*) and nine tumour suppressors (*AGTR1*, *DKK1*, *DPP4*, *EDNRB*, *GALR1*, *IGF1*, *IGF2R*, *KISS1*, and *NGFR*). The GALR, IGF and IGF receptor families have both oncogenic and tumour suppressing roles. As discussed in the literature, several cancer genes have dual roles in cancer progression depending on cancer type and the presence or absence of regulatory protein partners^18^. The dual role in the neuropeptide signaling pathways may provide the switch on cancer progression at the endocrine system level. In summary, our systematic examination of functional roles of 226 neuropeptides and receptors may uncover the regulatory roles of these endocrine signaling systems in cancer progression with both tumour suppressing and promoting effects.

### The module of the interaction map for neuropeptides and receptors

**Figure 3:**
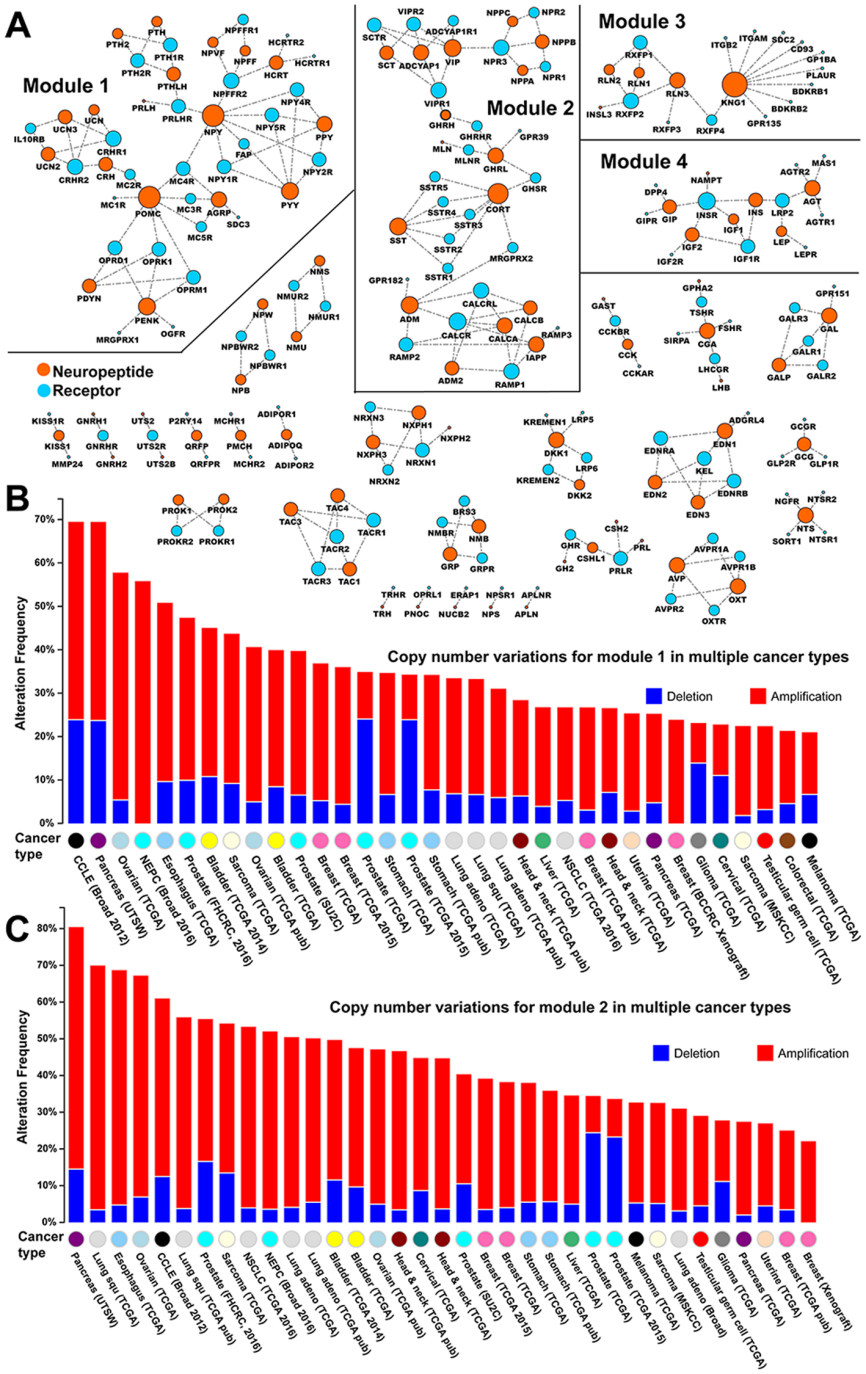
Functional modules in the interaction map for the 93 neuropeptides and 133 receptors. (**A**) The 93 genes in orange are neuropeptides. The other 133 receptor genes are in blue. The links between any two nodes represent the interactions. The size of the nodes represents the number of the connections. The top four connected modules are separated. (**B**) The global copy number variation for all the 44 genes in module 1 across multiple cancer dataset. (**C**) The global copy number variation for all the 39 genes in module 2 across multiple cancer dataset.

To further improve the systems level understanding of interactions between neuropeptide and receptors, we used the human ligand-receptor network^10^. These reliable ligand-receptor interactions are based on literature evidence, which is useful for the reconstruction of an accurate neuropeptide-receptor interactome and avoids the high level of inaccurate physical interaction-based protein-protein interaction data. We obtained the first neuropeptide-receptor interaction map (Fig. 3A). Due to the limitation of current known ligand-receptor data, some interaction pairs are separated from each other. For those connected neuropeptide-receptor pairs, we found a large number of modules. As shown in Fig. 3A, we highlighted the top four modules with the most interaction pairs. For module 1, we found a total of 44 genes, including 18 neuropeptides and 26 receptors, of which the *NPY* and *POMC* are the most connected neuropeptides with eight receptor links. These two neuropeptides share one common receptor, *MC4R*; MC4R dysfunction is a cause of autosomal dominant obesity^19^. In module 2, the most connected neuropeptide is *CORT* that has a function in inducing slow-wave sleep^20^. Although the neuropeptide *GHRN* only has two links in the module, it helps to bridge some separated neuropeptide signaling systems in module 2. Module 3 is centred by *KNG1*, which encodes multiple isoforms with blood coagulation, and antibacterial and antifungal activities^21^. Module 4 contains the *IGF* family and its interaction with *IGFR* receptors.

By incorporating the copy number variation (CNV) data of multiple cancers, we explored the CNV pattern for neuropeptides and receptors. By investigating the two biggest modules, we found more than ten cancer types with mutational frequency over 50% in 100 or more tumour samples. As shown by the CNV mutational pattern across multiple cancers plotted in Fig. 3B, module 1 was highly mutated in five cancer studies: CCLE, pancreatic, ovarian, neuroendocrine prostate, and oesophagus cancers ( > 50% samples). All the 44 genes in module 1 are identified as mutated in the CCLE dataset (Figure S2). Although most of the samples are mutated with amplification, the most frequently mutated gene is *MRGPRX1* with frequent deletion. As an orphan receptor, *MRGPRX1* can regulate nociceptor function to modulate sensation and pain^22^. Similarly, in the pancreatic cancer data set, we found that the receptor *MC4R* was deleted in 21 samples (Figure S3). For the module 2, we found consistent amplification for those top mutated genes in different cancer studies (Fig. 3C). In total, we found eleven dataset with mutational frequency greater than 50% in 100 or more tumour samples for module 2. For example, the top mutated neuropeptide *SCT* (secretin) is amplified in 26 samples while deleted in five samples in pancreatic cancer (Figure S4). Secretin was first identified and described in 1902 as a gut duodenum-derived chemical factor to stimulate pancreatic secretion^23^. Similarly, the genes in module 3 and 4 are also highly mutated in a few cancer types, including oesophagus, lung squamous cell, neuroendocrine prostate, ovarian, and pancreatic cancers. The systematic checking of the CNV for neuropeptide-related functional modules may indicate the importance of the neuropeptides in the cancer progression via the copy number change.

**Figure 4:**
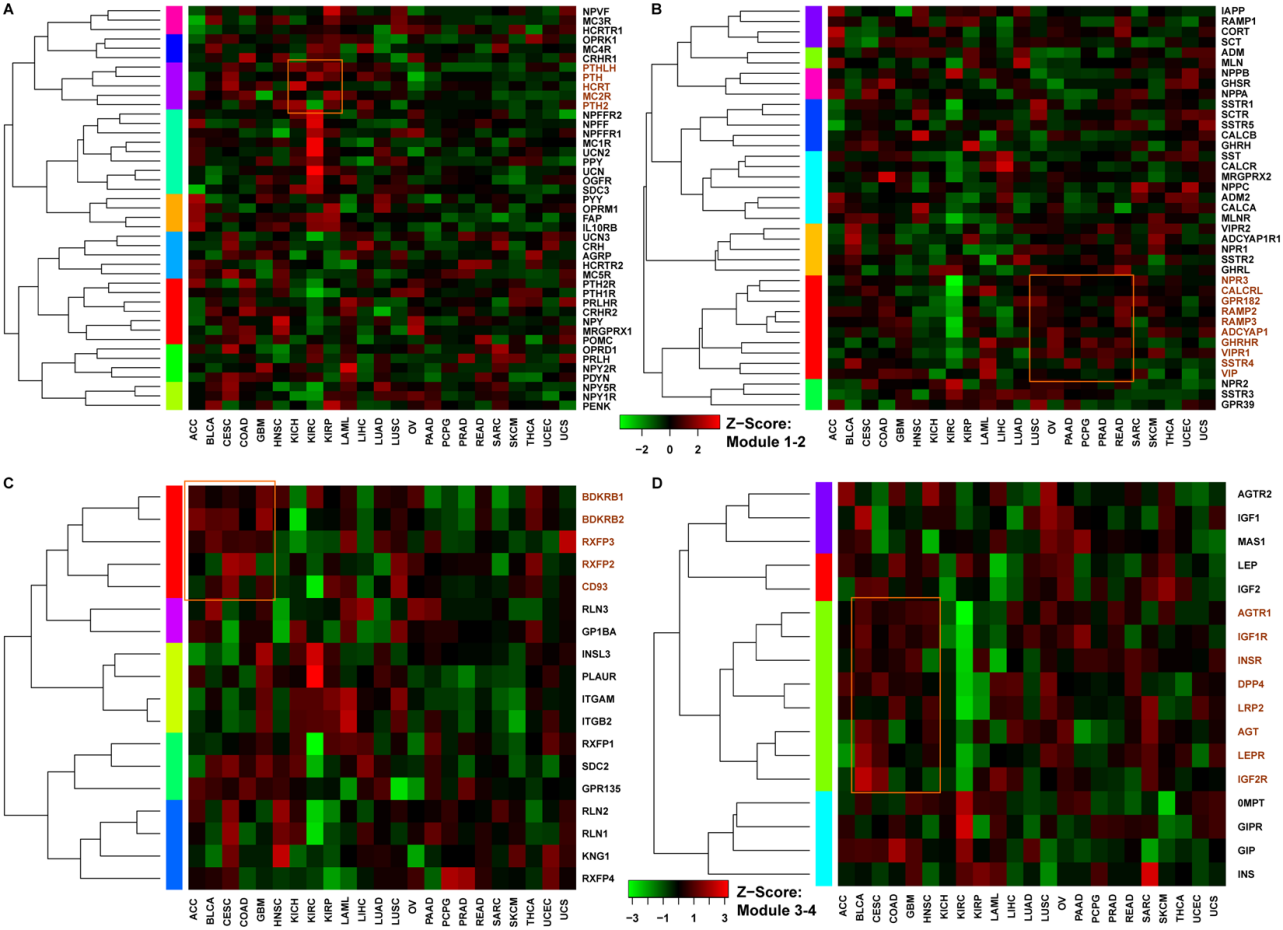
Heatmap of prognostic Z-scores of four neuropeptide-receptor modules in the 23 TCGA cancer set. The heatmap for module 1 (**A**), module 2 (**B**), module 3 (**C**), and module 4 (**D**) are presented with clusters based on the Z-score. The same scale of the Z-score was used for module 1 and 2 while the other scale of Z-score was used for module 3 and 4. The dendrograms for genes were shown on the left of each heatmap. The prognostic Z-scores are represented by the scale bar. Those genes within prognostic cluster are highlighted in dark yellow. The TCGA cancer types are abbreviated as ACC: Adrenocortical carcinoma; BLCA: Bladder Urothelial Carcinoma; CESC: Cervical squamous cell carcinoma and endocervical adenocarcinoma; COAD: Colon adenocarcinoma; GBM: Glioblastoma multiforme; HNSC: Head and Neck squamous cell carcinoma; KICH: Kidney Chromophobe; KIRC: Kidney renal clear cell carcinoma; KIRP: Kidney renal papillary cell carcinoma; LAML: Acute Myeloid Leukemia; LIHC: Liver hepatocellular carcinoma; LUAD: Lung adenocarcinoma; LUSC: Lung squamous cell carcinoma; OV: Ovarian serous cystadenocarcinoma; PAAD: Pancreatic adenocarcinoma; PCPG: Pheochromocytoma and Paraganglioma; PRAD: Prostate adenocarcinoma; READ: Rectum adenocarcinoma; SARC: Sarcoma; SKCM: Skin Cutaneous Melanoma; THCA: Thyroid carcinoma; UCEC: Uterine Corpus Endometrial Carcinoma; UCS: Uterine Carcinosarcoma.

We also examined the prognostic features of each module by using the PRECOG prognostic Z-scores across 23 TCGA cancer types. Those genes from the four functional modules were clustered into groups in the heatmap, which may reflect similar biological functions or effects on the tumorgenesis (Fig. 4). For module 1, a number of genes contain a high prognostic Z-score in KIRC (Fig. 4A). Also, five parathyroid hormone-related proteins (PTHrPs) (*PTHLH*, *PTH*, *HCRT*, *MC2R*, and *PTH2*) are prominent for three kidney cancers (KICH, KIRC, and KIRP). A recent study in 2006 confirmed that an anti-PTHrPs treatment strategy is a rationale for human kidney cancer growth without any side effects for body weight and blood chemistries^24^. We also found a large cluster of ten genes with potential prognostic application in module 2 for six cancers (Fig. 4B). Three pancreatic cancer types are in this cluster which suggests that these neuropeptides and receptors may be useful for prognosis of pan-pancreatic cancer. For example, two genes in this cluster (*RAMP2* and *RMAP3*) belong to the receptor activity-modifying protein (RAMP) family, which plays an important role in maintaining a healthy body weight in old age and may also be involved in immune function^25^. Similarly, we found that *BDKRB1/2* and *RXFP2/3* in module 3 have high prognostic significance in five cancer types (Fig. 4C). Also, the cluster with *IGF1R* and *IGF2R* in module 4 may also be useful for prognostic biomarkers in five cancers (Fig. 4D). In summary, the neuropeptide-receptor modules with multiple statistically prognostic Z-scores may the important for the design of anti-cancer drugs based on neuropeptides.

### Amplification of neuropeptide and receptor genes in neuroendocrine prostate cancer

**Figure 5:**
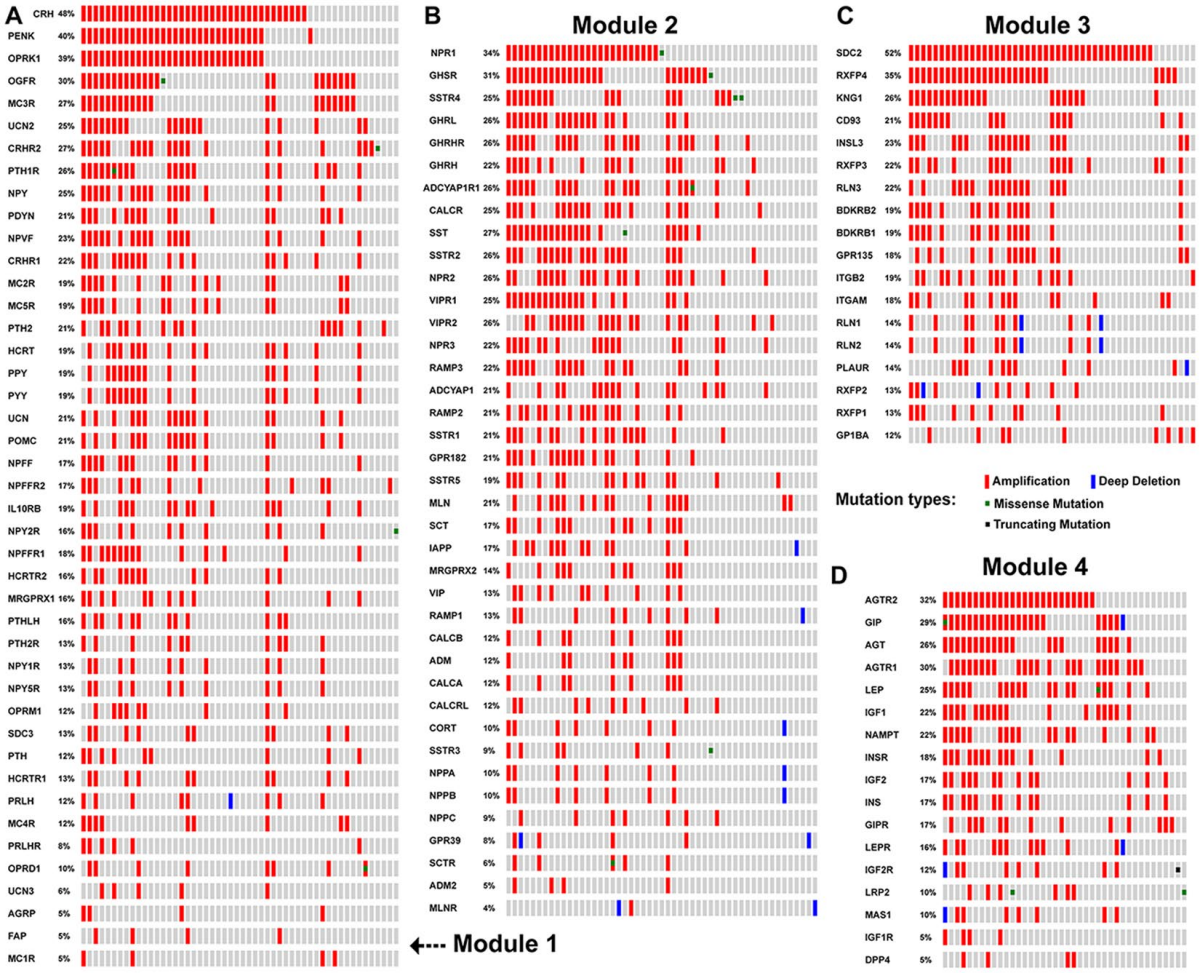
Somatic mutational patterns in neuroendocrine prostate cancer for the four modules. The sample-based mutational map for module 1 (**A**), module 2 (**B**), module 3 (**C**), and module 4 (**D**). The different mutational types are marked using different colours. The mutational types in (**A–D**) were depicted by colours. The red and blue represent the amplification and deep deletion respectively. The green and black dots are missense and truncating mutations respectively. The grey represents no mutations in the sample.

The copy number variation in genes could induce gene expression change by dosage effect^26^. To further examine the sample-based mutational pattern on neuropeptides and receptors, we focused on a neuroendocrine prostate cancer study. This research was to explore the genomics of those drug-resistant tumour samples with neuroendocrine features^27^. The four neuropeptide-receptor modules are consistent with dominant amplifications observed in the neuroendocrine prostate cancer study (Fig. 5). In total, the 118 genes from four neuropeptide-receptor modules were mutated in 81 out of 114 (72%) tumour samples. The *SDC2* in module 3 are amplified in 52% samples of the neuroendocrine prostate cancer cohort. This receptor belongs to the syndecan proteoglycan family that mediate cell proliferation, cell migration, cell-matrix interactions, and cytoskeletal organization^28^. The abnormal expression of *SDC2* has been detected in several different tumour types including melanoma^28^. We also found nine highly mutated genes with a mutational frequency of 30% or greater: *CRH* (48%), *PENK* (40%), *OPRK1* (39%), *RXFP4* (35%), *NPR1* (34%), *AGTR2* (32%), *GHSR* (31%), *OGFR* (30%) and *AGTR1* (30%). Seven of these genes co-occur with *SDC2* except *AGTR1* and *AGTR2* (P-values < 0.01, Table S4). For the evaluation of a potential biomedical application, we overlapped these 118 genes to drugable genes, immunome, secretome and surfaceome by using the OASIS analysis tool^29^. For all the 118 genes, we found 117 predicted to be drugable and 70 to be involved in immunological processes (Table S5). The protein products of two genes, *AGTR1* and *AGTR2*, have the highest durability score, which means they are candidate drug targets in terms of their biochemical features. It is not surprising that there are many genes associated with the secretome and surfaceome since all encode for neuropeptides and receptors. However, the 70 immunome-related genes may be useful for developing a new class of immune diagnostics by immunosequencing.

### The enrichment analysis of mutated genes against TCGA ovarian cancer mRNA expression and protein expression dataset for module 3 and 4

**Figure 6:**
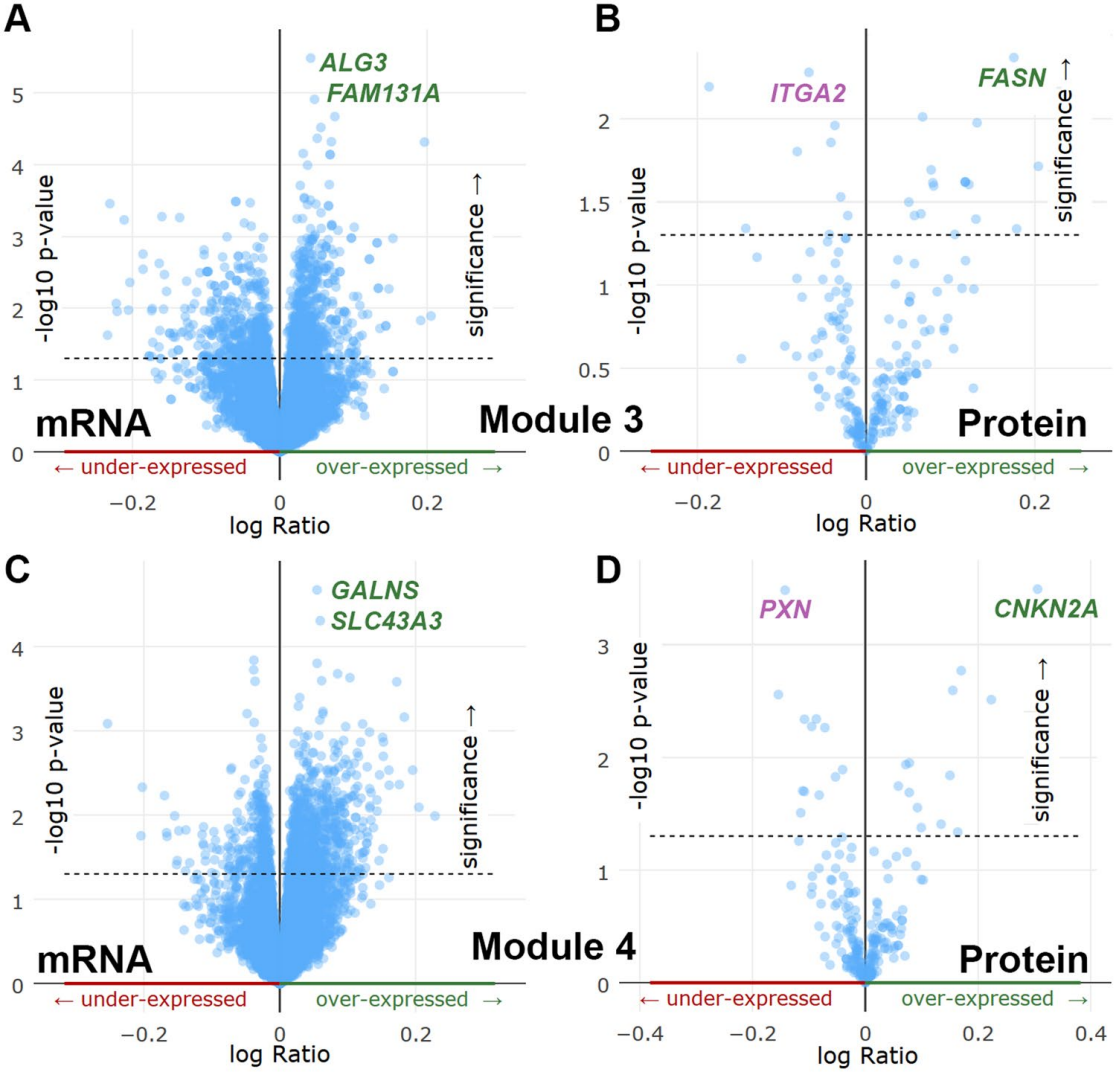
Enrichment analysis of mutated genes against TCGA ovarian cancer mRNA expression and protein expression dataset for module 3 and 4. Plots represent samples with mutated genes from module 3 overlapping to (**A**) gene expression and (**B**) protein expression in the same ovarian cancer samples. Plots represent samples with mutated genes from module 4 overlapping to (**C**) gene expression and (**D**) protein expression in the same ovarian cancer samples.

By using the gene expression and protein expression for those TCGA samples with mutated neuropeptide or receptors^16^, we tested whether samples with any mutated neuropeptides or receptors are enriched in those samples with high expression at the mRNA or protein level. By focusing on the TCGA ovarian cancer cohort mRNA and protein expression profiles, we explored those genes highly correlated with module 3 and 4. As shown in Fig. 6A, we found two genes *ALG3* and *FAM131A* that were highly expressed in those samples with gene mutated in module 3. The mean mRNA expression scores of *ALG3* are 11.46 and 11.13 in the mutated samples and non-mutated samples, respectively, which gave a corrected P-value of 0.0455 (Student test). These two genes belong to the same cytoband 3q27.1. We also found three more genes in the 3q27.1 with significant P-values less than 0.01, including *EIF2B5*, *ECE2*, and *ABCF3*. In summary, these results suggest that the samples with mutations on module 3 may have a relatively higher expression of those genes in 3q27.1, which could be caused by a co-regulatory mechanism. For the protein expression, we did not find any significantly differential expression after multiple testing corrections (all corrected P-values > 0.05). The two most differentially expressing genes are *FASN* and *ITGA2* (Fig. 6B). The fatty acid synthase (*FASN*) is crucial to meet the cancer cells increased demands for membrane via *de novo* long-chain fatty acid synthesis^30^.

Although those the enrichment analysis in mRNA expression of module 4 failed to identify significantly associated gene expression (all corrected P-values > 0.05), the result of protein level implicated two genes (*CDKN2A* and *PXN*, all corrected P-values < 0.05). In those samples with mutations in module 4, the mean expression of *CDKN2A* is 1.27, compared to 0.97 in those samples without any mutations. The *CDKN2A* is a tumour suppressor involved in multiple cancers and controls the cell cycle and proliferation^18^. In contrast, there was no *PXN* expression at the protein level in those samples with mutations in module 4. Paxillin (*PXN*) encodes a cytoskeletal protein involved in actin-membrane attachment and its expression could be used for clinical prognosis in colorectal cancer^31^. These sample-based enrichment results in module 4 may indicate the potential mechanisms of module 4-correlated gene expression change of *CDKN2A* and *PXN*.

## Conclusion

In conclusion, our systematic data collection of 127 human neuropeptides and 133 receptors provides the first comprehensive interactome of neuropeptides. By overlapping this information with cancer genomics data, we identified a number of promising neuropeptides and receptors as prognostic biomarkers in various cancers.

Our study is the first systematic examination of the relationships between human neuropeptides and the receptor interactome. By overlapping the interactome with large-scale cancer genomic data, we revealed several important somatic mutational features of neuropeptides and their receptors in multiple cancer types, particularly with respect to the CNV. Since scale change of gene copy number may induce changes in gene expression, the CNV events in neuropeptides and receptors may be critical drivers for cancer initiation and progression. We also explored the prognostic features of neuroeptide signalling in 23 TCGA cancer dataset because neuropeptides and their receptors may provide novel hormone-based therapeutic treatment with minimal side effects. By using enrichment analysis on ovarian cancer samples, we identified a number of co-expressed genes at both the RNA and protein level. Overall, our results provide the first insight of a correlation between the neuropeptide system and clinical survival outcome.

Therefore, the mutational pattern in this study was primarily based on the TCGA CNV data. However, the cohort size of those TCGA sets are relatively small with hundred individuals, which may filter out some potential low-frequency CNVs. Additionally, the current CNV data are mainly generated by the CGH array between normal and tumor tissue. Compared to the NGS-based approach, the CNVs outside of pre-designed probes were undetected, but these may also relate to neuropeptide and their receptors functionality in cancer development. Another limitation of this study is that we only incorporated the gene expression-based prognostic features, not a combination of mutation and expression. The further integration of large-scale mutational data and gene expression of neuropeptide and receptors may provide new insight into the roles of the neuropeptides in different cancers.

## Methods

### Curation of neuropeptides from multiple data sources

To survey the role of neuropeptide in human cancers, we performed an extensive database and literature search. There were three data sources for human neuropeptides used in this study: keyword-based searching against NCBI RefSeq protein database; a comprehensive database of neuropeptides NeuroPep^32^; and two published manuscripts concerning neuropeptide evolutionary analysis^33, 34^. By removing the redundant genes and updating the gene symbols, we have identified 127 human genes that encode neuropeptide precursors (Table S1). These precursor genes are usually processed to produce hundreds of neuropeptide by alternative splicing and post-translational modification^2^.

### Cancer-specific gene databases

To study the cancer-specific roles of the neuropeptides and receptors, we used five cancer-specific gene databases^11–14^, including gbCRC for colorectal cancer, ECGene for endometrial cancer, OCGene for ovarian cancer, and PCGene for pancreatic cancer. All the gene entries in these databases are based on extensive data integration and literature curation. The data integration strategy was to integrate those cancer-specific genes resources such as OMIM and GWASCatalog. The literature search focused on each cancer by using a regular expression against GeneRif database: taking PCGene as example, [(pancreatic) AND (cancer OR tumor OR carcinoma)]. Finally, those experimentally verified candidate genes were manually assembled and annotated using public cancer genomics data such as single-nucleotide variations and gene expression.

### TCGA pan-cancer prognostic data from PRECOG

To explore the prognostic feature in pan-cancer systematically, we downloaded all the precomputed TCGA prognostic Z-score data from PRECOG^9^. By integrating multiple cancer expression and ENCODE data, the PRECOG provided global prognostic features for all the human genes across 39 malignancies. By focusing on TCGA gene-level RNA-seq and survival data, PRECOG associate of each gene with survival outcomes by Cox proportional hazards regression. The standard score Z was used to characterize whether a gene is associated with significant longer or shorter survival time. The Z-score over 1.96 is defined as statistically prognostic indicative for long survival while the Z-score less than −1.96 indicates the significantly decreased survival time.

The heatmaps of the Z-score were visualized using the “heatmap” function in R language. The clustering of the Z-score was conducted using the “hclust” function in R language. The similarity matrix to build clusters was based on the person distance of those input values. The agglomeration method to find similar clusters was “complete linkage.”

### Neuropeptide-receptor network construction and visualization

To explore the downstream signalling transduction pathway of neuropeptides, we extracted a total of 260 neuropeptide-receptor pairs from a human ligand-receptor network^10^. This network has examined detailed transmembrane and secreted proteins specifically involved in cell-to-cell communication by integrating literature and computational data. In total, there are 1,894 known ligand-receptor pairs with literature evidence and 528 putative interactions with co-localized and functional relevance.

We extracted 260 neuropeptide-receptor pairs with 93 neuropeptides and 133 receptor genes (Table S2). Based on the neuropeptide-receptor interactions, we connected all the 226 genes with 260 links. By checking those fully connected subnetworks, we identified four functional modules each with at least ten genes. The network layout was conducted based on Cytoscape 2.8^35^. For those genes in the network, we further systematically examined their somatic mutational pattern such as CNVs in pan-cancer of TCGA samples using cBio portal^36^.

## Electronic supplementary material

Supplementary figures

Table S1

Table S3

Table S4

Table S5

Table S2
