# Supplementary material for: Copy number alteration of neuropeptides and receptors in multiple cancers
Source: Sci Rep. 2017 Jul 4;7:4598. doi: 10.1038/s41598-017-04832-0 (PMC5496884; doi:10.1038/s41598-017-04832-0)

Copy number alteration of neuropeptides and receptors in multiple cancers

Min Zhao[1§], Tianfang Wang[1], Qi Liu[2,3], Scott Cummins[1§]

[1]School of Engineering, Faculty of Science, Health, Education and Engineering, University of the Sunshine Coast, Maroochydore DC, Queensland, 4558, Australia.

[2]Department of Biomedical Informatics, Vanderbilt University School of Medicine, Nashville, Tennessee 37232, United States.

[3]Center for Quantitative Sciences, Vanderbilt University School of Medicine, Nashville, Tennessee 37232, United States.

[§] To whom correspondence should be addressed, mzhao@usc.edu.au, scummins@usc.edu.au.

Supplement

Table S1. Details of 127 human genes that encode neuropeptide precursors.

Table S2. Prognostic Z-scores of human neuropeptide across 23 cancer types.

Table S3. Interaction of 93 neuropeptides and 133 receptors.

Table S4. Co-mutated genes with SDC2 in neuroendocrine prostate cancer.

Table S5. Global feature of 118 genes from four functional modules.

Figure S1. Scatterplot about the cellular component (A) and molecular function (B) for 127 neuropeptides.

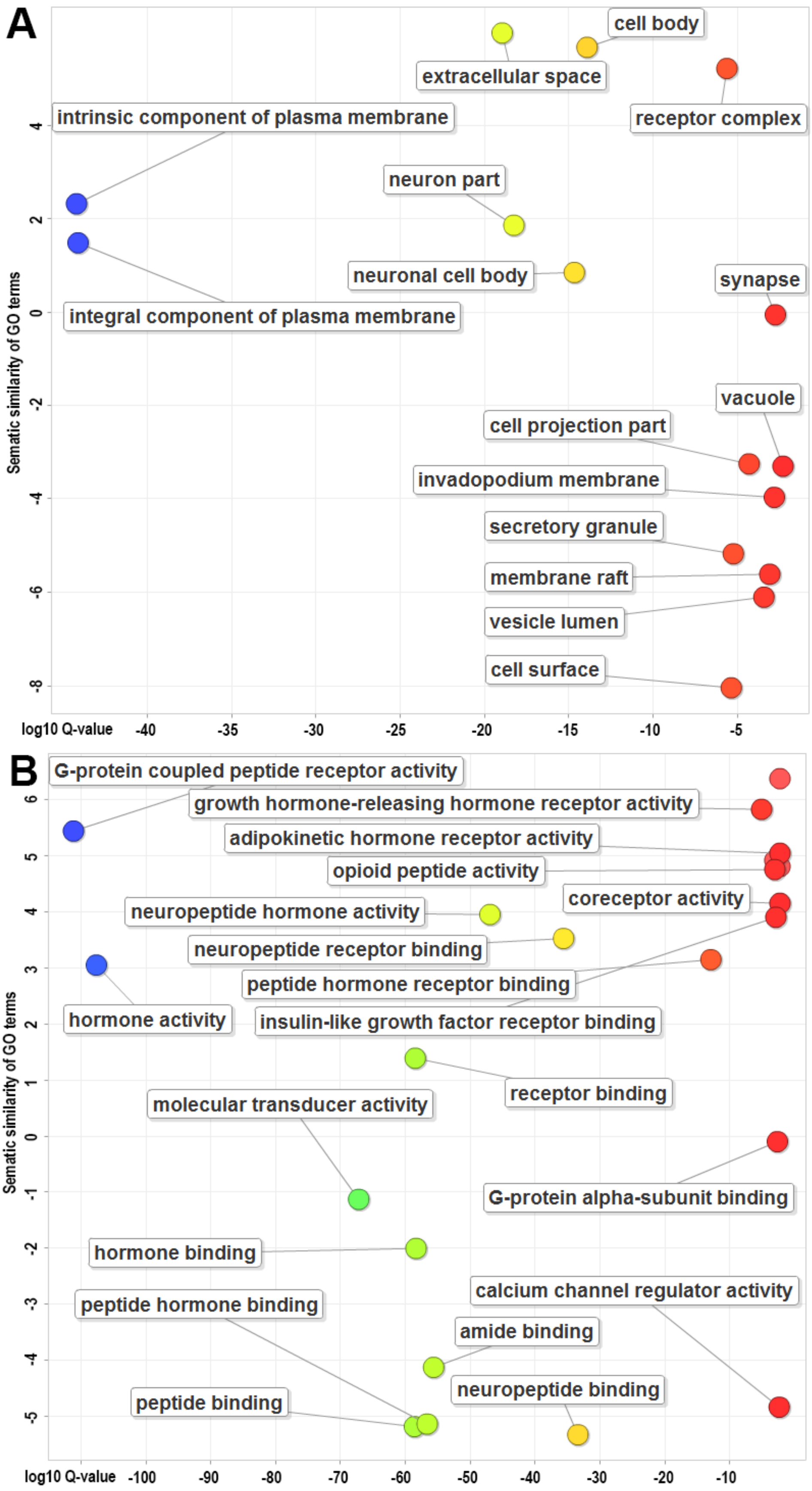

Figure S2. Mutational pattern of 44 genes in module 1 for CCLE dataset.

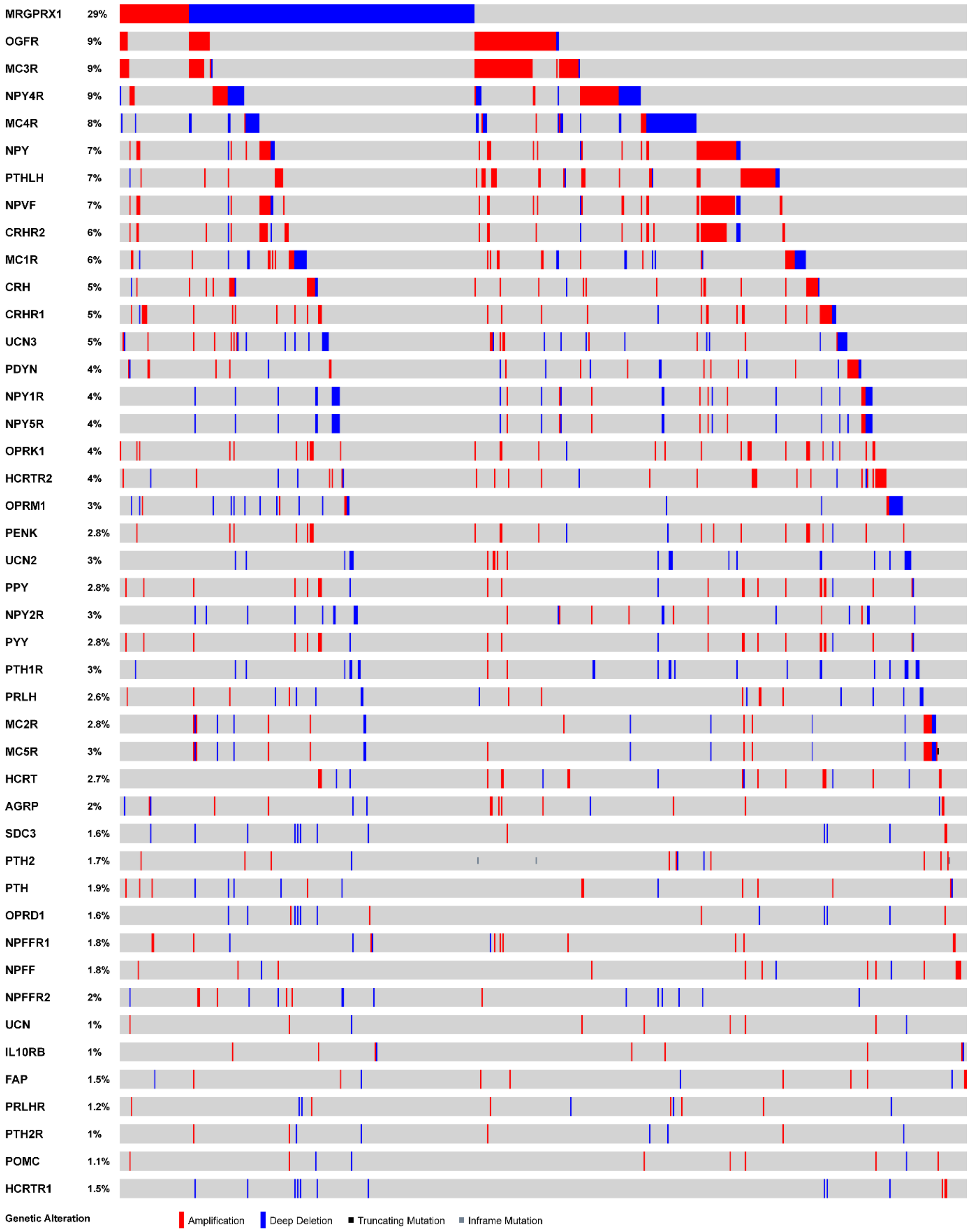

Figure S3. Mutational pattern of 44 genes in module 1 for pancreatic cancer.

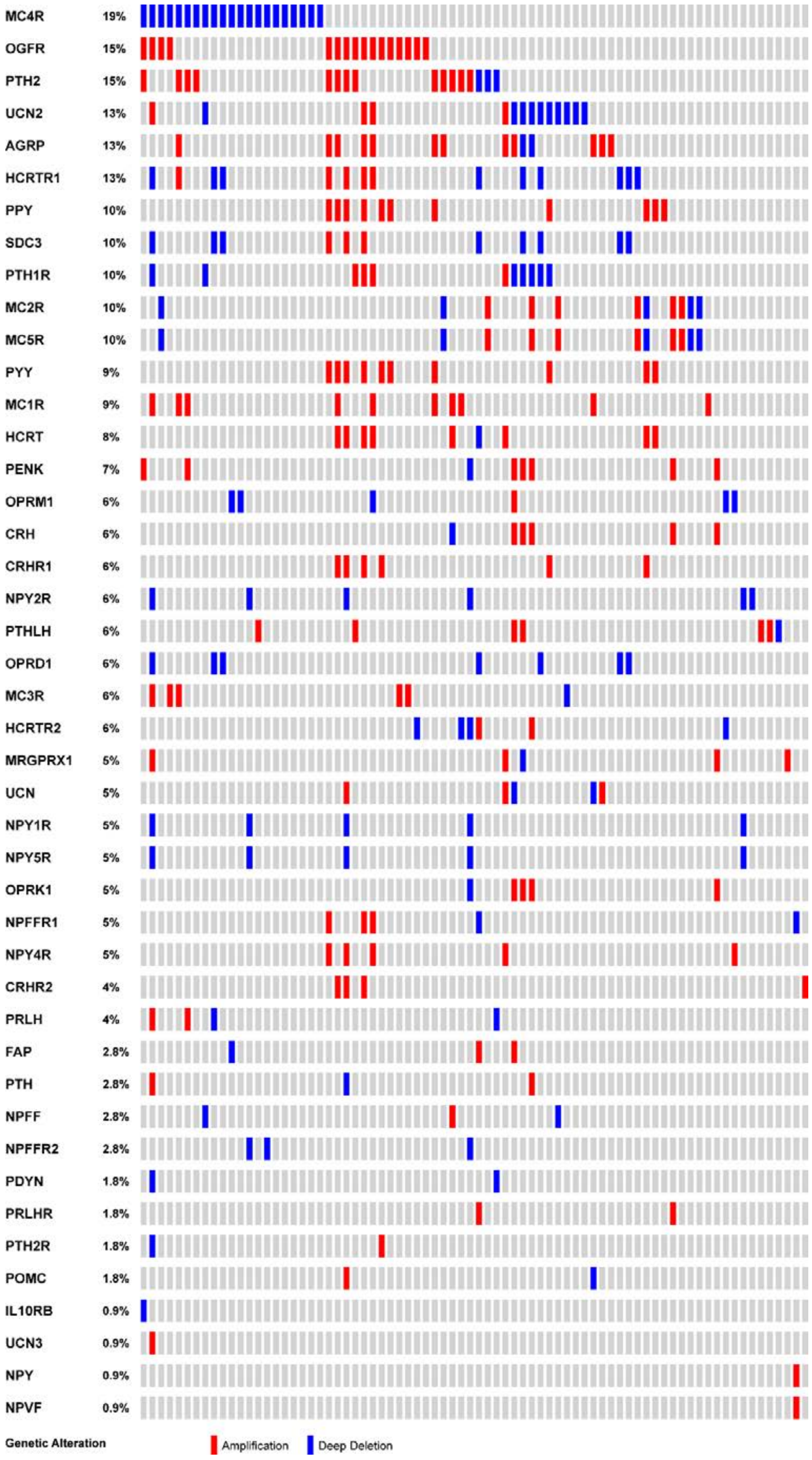

Figure S4. Mutational pattern of 39 genes in module 2 for pancreatic cancer.

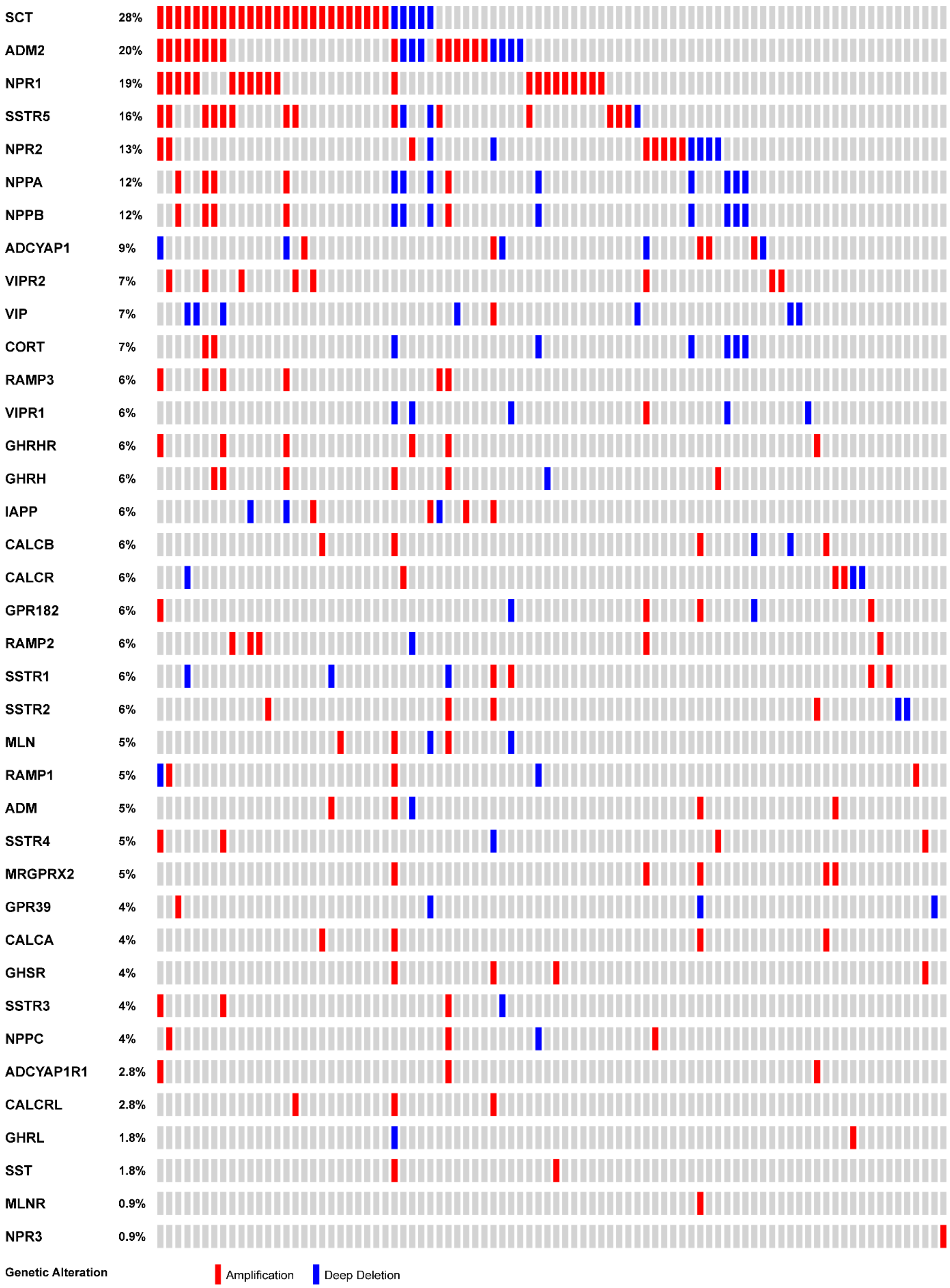

Supplement: Supplementary file 1 — Supplementary figures [file 41598_2017_4832_MOESM1_ESM.pdf]
